# A clinical study of microwave ablation for cervical lymph node metastasis from papillary thyroid cancer

**DOI:** 10.3389/fendo.2025.1580765

**Published:** 2025-04-25

**Authors:** Jie Wu, Ying Wei, Zhen-long Zhao, Shi-liang Cao, Yan Li, Li-li Peng, Shu-qi Li, Ming-an Yu

**Affiliations:** China-Japan Friendship Hospital, Beijing, China

**Keywords:** cervical lymph node metastasis, papillary thyroid cancer, microwave ablation, tumor recurrence and metastasis, minimally invasive treatment

## Abstract

**Purpose:**

The aim of this study is to evaluate the efficacy and safety of microwave ablation (MWA) for cervical lymph node metastasis (LNM) in initially treated, post-ablation and post-resection papillary thyroid cancer (PTC) patients.

**Methods:**

A total of 131 patients with 535 LNM from PTC who underwent ultrasound-guided MWA were included in the retrospective study. Patients were divided into three subgroups on the basis of treatment timing: initially treated, after PTC ablation (post-ablation), or after resection (post-resection). Changes in cervical metastatic lymph nodes as well as the incidences of complications, tumour recurrence and progression were compared.

**Results:**

The technical success rate of this study was 100% (535/535). Compared with those before MWA, the mean largest diameter and volume of the metastatic lymph nodes were significantly lower (p <0.01) at each follow-up. Transient hoarseness was the exclusive major complication with a total rate of 5.3% (9/171), which significantly differed in terms of incidence among the three subgroups (p<0.01). Lymph node location in region VI was an independent risk factor for transient hoarseness. The total recurrence rate was 22.8% (39/171) without statistically significant difference among the three sub-groups (p=0.20). Two cases received repeated surgery, while re-ablation was conducted successfully in all rest of cases. Data from the latest follow-up revealed one death due to LNM.

**Conclusion:**

MWA is a safe and effective treatment option for LNM in initially treated, post-resection and post-ablation PTC patients.

## Background

Papillary thyroid carcinoma (PTC) represents approximately 80–85% of all thyroid cancer cases and poses significant challenges in thyroid cancer treatment (1–3). This malignancy predominantly metastasizes via the lymphatic system, generally affecting the central compartment of the cervical lymph nodes before progressing to the lateral compartments, with 20–90% of central lymph node metastasis (LNM) cases reported in PTC patients (4, 5). Although controversial, prophylactic central lymph node dissection is frequently performed in PTC patients with clinically negative central lymph nodes (CN0) (6).

Moreover, a notable proportion of patients (5–20%) experience recurrences and LNM postoperatively (7). According to the 2015 American Thyroid Association guidelines, reoperation and/or radioiodine ablation is recommended for PTC patients with LNM (6). Repeat surgeries for LNM are often complicated and challenging owing to postoperative fibrosis and tissue deformation, increasing the risk of serious complications, including laryngeal nerve paralysis and hypoparathyroidism, and impairing quality of life (8–10). Therefore, a minimally invasive technique is required as a therapeutic alternative to surgical resection.

Recent developments in ultrasound-guided thermal ablation (TA), including microwave ablation (MWA) and radiofrequency ablation (RFA), have preliminarily demonstrated promising outcomes and a low incidence of complications in treating LNM in PTC patients (11–16), which have been recommended in relevant guidelines (6, 17). However, comprehensive research on the efficacy and safety of thermal ablation in PTC patients with LNM is still in progress.

Therefore, this study aims to evaluate the efficacy and safety of MWA for treating LNM in PTC patients, including initially treated and after PTC ablation (post-ablation), or after resection (post-resection).

## Materials and methods

### Patients

This retrospective study, conducted at the China–Japan Friendship Hospital (Beijing, China) between November 2015 and November 2022, included 131 patients with 535 LNM from PTC, treated with microwave ablation (MWA). Inclusion criteria were: (1) LNM from PTC confirmed via fine-needle aspiration biopsy (FNAB) before ablation; (2) refusal of neck dissection; (3) follow-up exceeding 9 months. (4) the maximum diameter of LNM ≤ 5cm. Exclusion criteria included: (1) cases under 16 years old or pregnant women; (2) distant metastasis; (3) serious bleeding tendencies; (4) LNM with severe adhesion or invasion to surrounding structures, making successful hydrodissection impossible. The institutional ethics committee approved this study, and the requirement for patient consent was waived due to the retrospective nature of the study.

Patients were divided into three subgroups according to the timing of treatment: initially treated, post-ablation and post-resection. The initially treated subgroup included PTC cases with LNM underwent MWA for both PTC and LNM simultaneously. The post-ablation subgroup included cases with recurrent LNM following previous ablation for PTC. The post- resection subgroup included PTC cases with recurrent LNM after prior surgical resection.

### Equipment

GE LOGIQ E9 (GE Healthcare, USA) systems equipped with a 9.0 MHz linear probe were utilized for the US examination and guidance. A microwave generator and a 17-gauge internally cooled antenna (Nanjing ECO Microwave System, China) were used for the ablation procedure. Sonovoxes (Bracco, Italy) was the US contrast agent, administered in a 2.0 ml bolus followed by a 10 ml saline flush (18).

### Operators and MWA procedure

FNAB and MWA procedures were performed by radiologists with more than three years of thyroid imaging experience. Patients were positioned supine with fully exposure of neck. The ablation site was sterilized and draped with sterile towels. Pre-MWA CEUS was conducted in all patients to evaluate the enhancement pattern of target LNM. Local anesthesia (1% lidocaine) was administered, followed by the injection of isolation fluid (0.5% lidocaine in normal saline, 1:3 ratio) around the target LNM. The 18G PTC needle tip was placed inside the fascia surrounding the LNM to achieve circular hydrodissection wrapping around the LNM. If necessary, multiple PTC needles could be applied. To prevent heat injury to surrounding critical structures, isolation fluid was continuously injected during radiation to guarantee safe separation distance for at least 4mm.

Specifically, hydrodissection was strategically applied depending on the LNM’s location. For target LNM near the trachea or esophagus, isolation fluid was injected to lift the LNM anteriorly, protecting the recurrent laryngeal nerve, trachea and esophagus from heat damage. When targeting LNM near or within the carotid sheath, the isolation fluid was injected into the carotid sheath to safeguard the vagus nerve, carotid artery, and internal jugular vein.

**Figure 1:**
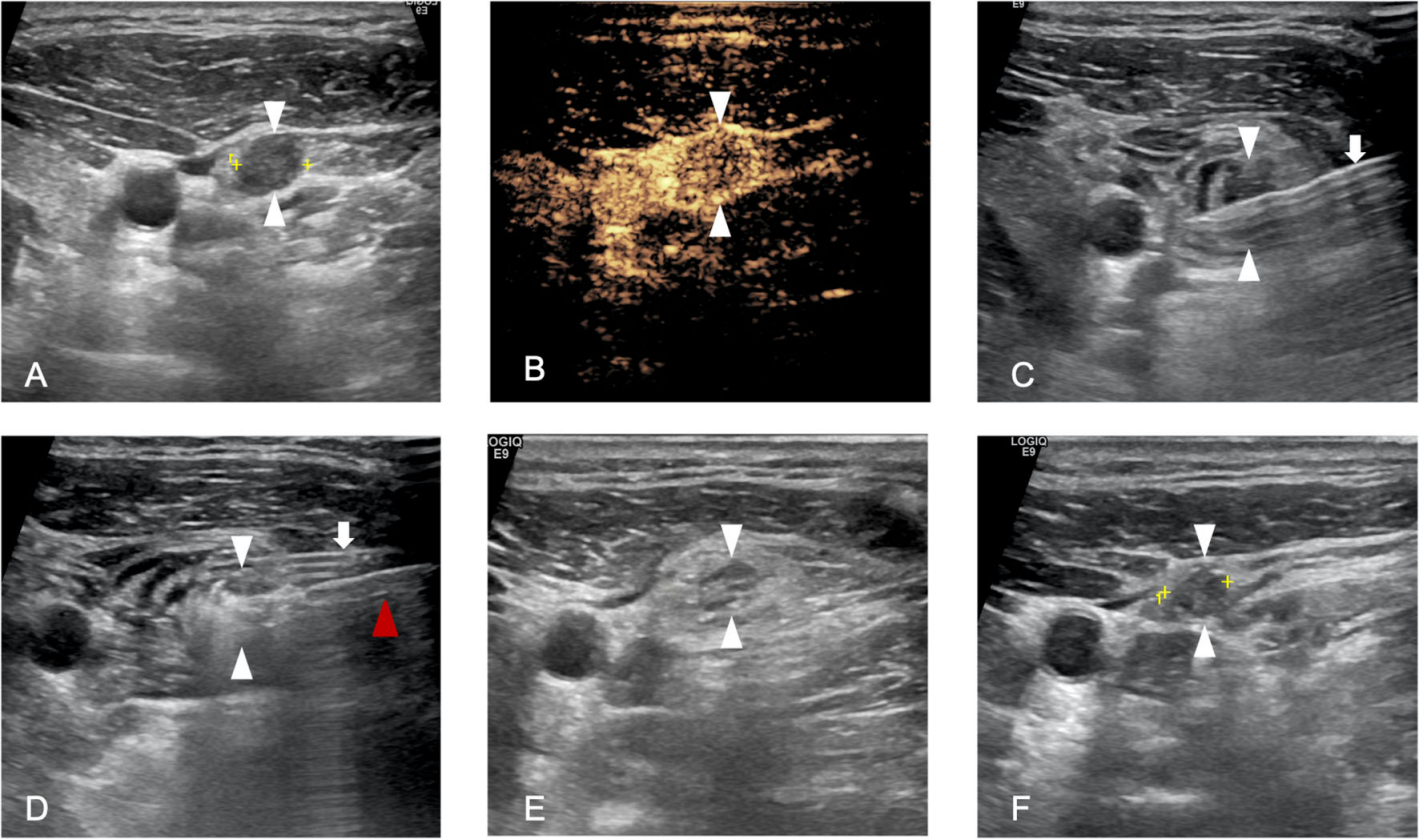
A 36-year-old man who underwent microwave ablation (MWA) of cervical lymph node metastasis (LNM). **(A)**, Pre-MWA, B-mode ultrasonography (US) showed hypoechoic LN (white arrowhead); **(B)**, Pre-MWA, the contrast-enhanced US (CEUS) showed uneven enhanced patterns (white arrowhead); **(C)**, Hydrodissection fluid was injected surround the LNM (white arrowhead) by 18G PTC needle(white arrow); **(D)**, Hyperechoic (white arrowhead) pattern in the LNM during MWA, and red arrowhead refers to MWA antenna; **(F)**, One day post-MWA, hyperechoic ablated needle tract was showed inside the LNM (white arrowhead); **(F)**, One month post-MWA, the size of LNM decreased compared to pre-MWA.

In the MWA procedure, the needle tip of the 17 G MWA antenna was inserted into the target lesion under US guidance. The power was 30 W-35W. Based on the tumor characteristics, the moving-shot, multi-point, or fixed applicator technique was applied according to the size. The ablation was terminated when a transient hyper echo was observed throughout the target LNM. Post-MWA, CEUS was conducted to evaluate the effect of ablation. Complete ablation was determined when the entire LNM showed no enhancement. If any enhanced area was detected within the target LNM, additional ablation was performed immediately. After the complete ablation of one LNM, the MWA procedure was repeated for subsequent LNM. The entire procedure including pre-ablation B-mode US, CEUS examination, ablation process, and post-ablation follow-up examination are illustrated in **Figure 1**. For patients with multiple LNM, planned staged ablation can be applied.

### Post-ablation follow-up

Technical success was determined by the complete absence of enhancement in the target LNM on CEUS (12). Complication assessment and recordation were conformed to the standards for image-guided thyroid ablation (19). Follow-up was scheduled quarterly in the first year, biannually (every 6 months) in the second year, and annually thereafter. Detailed data from patient examinations at each follow-up were systematically recorded. Each follow-up evaluation involved blood tests, and US examination to monitor for recurrence. CT examination was conducted to evaluate distant metastasis annually.

For the ablated area, a routine neck B-mode Ultrasound (US) examination was performed. Contrast-Enhanced Ultrasound (CEUS) and FNAB were conducted when local recurrence or new LNM was suspected. Dimensions of the target LNM were measured during the US examination. The volume of the LNM was calculated using the formula: V=πabc/6, where *V* represents the volume, and ‘a’, ‘b’, and ‘c’ denote the largest diameter and the two other perpendicular diameters, respectively (20).

### Statistical methods

Statistical analyses were performed using SPSS version 24.0 (IBM, Armonk, NY, USA). The data are presented as the mean ± standard deviation (SD), or the median and 25-75% interquartile range (IQR) were used if data did not fit a normal distribution. *P*<0.05 was considered to indicate statistical significance.

The Wilcoxon signed rank test was used to compare the changes in the size and volume of LNM before and after ablation. The recurrence rates and complication rates in the initially treated, post-resection and post-ablation subgroups were compared by Fisher’s exact test. Risk factors predicting complications were analyzed using univariate analysis and multivariate logistic regression analysis. Technical feasibility and technical success were evaluated. During follow-up, tumour disappearance, local recurrence, new LNM, distant progression and complication rates were also evaluated.

## Results

### Patient characteristics

The data of 131 patients (age range 16–88 years, mean ± SD: 42.40 ± 14.04 years) enrolled in present study are summarized in **Table 1**. There were 45 men (34.4%) and 86 women (65.6%). For the number of primary PTC tumors, 93 patients (71.0%) had solitary tumor, 24 patients (18.3%) had two tumors, 14 patients (10.7%) had multiple tumors (≥3). Among these patients, 32 (24.4%) had undergone once surgical resection, 3 (2.3%) had undergone twice surgical resections, 96 cases have no history of surgical resection. Among them, 14 cases (10.7%) have undergone one session thermal ablation for thyroid cancer, 82 cases (62.6%) choose thermal ablation for treating PTC and LNM for the first time. The number of LNM was 1 to 9, with an average of 4 LNM per case. Follow-up durations varied from 9 to 105 months, with the mean follow-up time of 45.02± 18.30 months.

**Table 1:** Clinical characteristics of the study population.

Variables	Total
Age (years)*	42.40 ± 14.04
Male	45 (34.4%)
Female	86 (65.6%)
Follow-up (months)*	45.02± 18.30
Number of primary PTC tumors	
Solitary tumor	93 (71.0%)
Right lobe	49 (37.4%)
Left lobe	42 (32.1%)
Isthmus	2 (1.5%)
two tumors	24 (18.3%)
Both right lobe	3 (2.3%)
Both left lobe	3 (2.3%)
Right lobe and left lobe	13 (9.9%)
Isthmus and right lobe	3 (2.3%)
Isthmus and left lobe	2 (1.5%)
Multiple tumors (≥3)	14 (10.7%)
ALL right lobe	1 (0.8%)
Right lobe and left lobe	9 (6.9%)
Right lobe, left lobe and isthmus	4 (3.0%)
Medical history	
None	82 (62.6%)
Post one session thermal ablation	14 (10.7%)
Post-surgical resection	35 (26.7%)
Once	32 (24.4%)
Twice	3 (2.3%)

Unless indicated otherwise, data are presented as frequencies (percentage). *Data presented as mean ± standard deviation (range).

### LNM characteristics

In **Table 2**, the characteristics of the LNM are summarized. Of the 535 LNM, 294 (55.0%) were in the left neck and 241 (45.0%) in the right neck. There were 21 (3.9%), 43 (8.0%), 129 (24.1%), 152 (28.4%), 20 (37.4%), 153 (28.6%) and 17(3.2%) LNM at levels I, II, III, IV, V, VI and VII, respectively. The pre-ablation largest diameter of LNM ranged from 2.0 to 48.0 mm (median 10.0mm, IQR8.0-16.0mm). The volume of the cervical metastatic LNM ranged from 4.2 to 40204.8 mm^3^ (median 230.9mm^3^, IQR114.8-722.0mm^3^). In the pre-ablation CEUS, LNM showed homogeneous enhancement in 101 patients, and inhomogeneous enhancement in 30 patients. The total ablation time of patients ranged from 8 to 688 s (median 74.5 s, IQR34.3-167.8s). The ablation time of LNM ranged from 4 to 417 s (median 36 s, IQR18.5-67.9s).

**Table 2:** The characteristics of the LNM.

Characteristics	N (%)
Left and right neck	
Left	294 (55.0%)
Right	241 (45.0%)
Location	
Level I	21 (3.9%)
Level II	43 (8.0%)
Level II	129 (24.1%)
Level III	152 (28.4%)
Level IV	20 (37.4%)
Level VI	153 (28.6%)
Level VII	17(3.2%)
Pre-ablation maximum diameter (mm)	
Range	2.0-48.0
Median	10.0
Interquartile range	8.0-16.0
Pre-ablation volume (mm^3^)	
Range	4.2 to 40204.8
Median	230.9
Interquartile range	115.4-723.2
Total ablation time per patient (seconds)	
Range	8–688
Median	74.5
Interquartile range	34.3-167.8
Ablation time per LNM (seconds)	
Range	4–417
Median	36.0
Interquartile range	18.5-67.9
Pre-MWA CEUS	
Homogeneous	101(77.10%)
Inhomogeneous	30(22.90%)

### Treatment outcomes and complications of MWA

In present study, complete ablation was achieved in all LNM, as evidenced by the absence of enhancement in post-ablation CEUS. The technical success rate was 100% (131/131).

Detailed changes in the largest diameter and volume of LNM pre- and post-ablation across all follow-up intervals, are depicted in **Table 3**. The median largest LNM diameters in subsequent follow-ups (1,3, 6, 9 months post-ablation) decreased progressively from 8.0mm to 5.0mm, 0.0mm, 0.0mm, respectively. Similarly, median LNM volumes reduced from 128.8 mm^3^ to 37.7 mm^3^,3.2 mm^3^, 0.8 mm^3^. These reductions in both diameter and volume at each follow-up were statistically significant (p <.05). There were 84.1% (450/535) of lesions completely disappearing in the last follow-up. 16 patients underwent staged ablation for two procedures, and 3 patients for three procedures.

**Table 3:** Changes in cervical metastatic lymph nodes post-ablation at each follow-up.

Follow-up time	Largest diameter (mm)	*p* value	Volume (mm^3^)	*p* value
Pre-thermal ablation	10.0 (8.0–16.0)	–	230.9(114.8–722.0)	–
1-month post-thermal ablation	8.0 (5.0–13.0)	<.001	128.8 (38.9–384.8)	<.001
3-month post-thermal ablation	5.0 (0.0–10.0)	<.001	37.7 (1.7–147.0)	<.001
6-month post-thermal ablation	0.0 (0.0–5.0)	<.001	3.2 (0.0–13.1)	<.001
9-month post-thermal ablation	0.0 (0.0–0.0)	<.001	0.8 (0.0–6.5)	<.001

The largest diameter and volume are expressed as median (Interquartile range). *p* value: post-ablation (3, 6, 9, 12, 18, 24, and 36 months) vs. pre-ablation, respectively.

During follow-up, there is no local recurrence or distal metastasis. The total recurrence rate was 22.8% (39/171) with the mean follow-up time of 45.02± 18.30 months (9-105 months). Detailed recurrence rates in three subgroups are depicted in **Table 4**. Specifically for subgroup analysis, the total recurrence rate was 24.4% (30/112) in initially treated subgroup, 6.7% (1/15) in post-ablation subgroup and 20.5% (9/44) in post-resection subgroup respectively. There was no statistically significant difference among the three sub-groups (*p*=0.20). For patients initially treated (26.7%, 30/112) and post-resection/ablation (16.9%, 10/59), there was no statistically significant difference (*p*=0.19). Especially for patients post-resection (20.5%, 9/44) and post-ablation (6.7%, 1/15), there was also no statistically significant difference (*p*=0.43).

**Table 4:** Recurrence rates and complication rates in three subgroups.

Subgroup	Case number	Recurrence rate	Complication Rate
Initially treated (82)			
First-time ablation	82	30.5% (25/82)	8.5% (7/82)
Second-time ablation	26 (included 2 staged ablations, another 1 received resection)	16% (4/25)	7.7% (2/26)
		26.9% (29/108) *	8.3% (9/108) *
Multiple-time ablation	4 (another 1 received resection)	26.7% (30/112) *	8.0% (9/112) *
Post-ablation (14)			
First-time ablation	14	7.1% (1/14)	0.0% (0/14)
Second-time ablation	1	0.0% (0/1)	0.0% (0/1)
		6.7% (1/15) *	0.0% (0/15) *
Post-resection (35)			
First-time ablation	35	17.1% (6/35)	0.0% (0/35)
Second-time ablation	6	16.7% (1/6)	0.0% (0/6)
		17.1% (7/41) *	0.0% (0/41) *
Multiple-time ablation	3	20.5% (9/44) *	0.0% (0/44) *

*Data were presented as accumulated total incidence rate for three subgroups.

For recurrence treatment, new LNM in 31 cases were treated successfully by second-time ablation, while one patient received total thyroidectomy and bilateral neck lymph node dissection due to new PTC and new LNM. Detailed recurrence rates after each time ablation are depicted in **Table 5**. However, five patients exhibited new LNM for the third time, included four patients (16%, 4/25) from initially treated subgroup, one patient (16.7%, 1/6) from post-resection subgroup and none (0.0%, 0/1) in post-ablation subgroup. There was no statistically significant difference among the three sub-groups for the recurrence rate after second-time ablation (p=1.0). Four patients underwent the third-time ablation successfully, while one patient received neck lymph node dissection due to new LNM. Then, one patient from initially treated subgroup exhibited another new LNM after 5 months from the third-time ablation, and received fourth-time ablation. There has been no recurrence as of the latest follow-up. There was another patient from post-resection subgroup exhibited new LNM after 14 months from the third-time ablation, and also received fourth-time ablation. However, this patient exhibited another new LNM after 4 months and received fifth-time ablation. Due to LNM, the patient ultimately died at the age of 78 years old 45 years after first PTC surgery.

**Table 5:** Recurrence rates and complication rates post each time ablation.

Ablation	Case Number	Recurrence Rate	Complication Rate
First-time ablation	131	24.4% (32/131)	5.3% (7/131)
Second-time ablation	33 (included 2 staged ablations, another 1 received resection)	15.6% (5/32)	6.0% (2/33)
		22.6% (37/164) *	5.5% (9/164) *
Multiple-time ablation	7 (another 1 received resection)	22.8% (39/171) *	5.3% (9/171) *

*Data were presented as accumulated total incidence rate.

Detailed complication rates in three subgroups and post each time ablation are depicted in **Tables 4**, **5**. Nine patients (9/171, 5.3%) experienced transient hoarseness post ablation, in which all patients underwent LNM ablation in region VI, and the symptoms were all automatically recovered within 6 months. Among them, seven patients experienced transient hoarseness for the first ablation (5.3%, 7/131), two patients for re-ablation (6.0%, 2/33) due to new LNM after once ablation of PTC and LNM. The rate of transient hoarseness showed no significant difference between first ablation and re-ablation(p>0.05). Specifically for subgroup analysis, there were nine patients (8.0%, 9/112) in initially treated subgroup, none (0.0%, 0/15) in post-ablation subgroup and none (0.0%, 0/44) in post-resection subgroup. There was statistically significant difference between the three subgroups (p<0.01). There was also statistically significant difference between the groups of initially treated (8.0%, 9/112) and post-resection/ablation (0.0%, 0/59) (p<0.05). For risk factors, the rate of hoarseness in ablating LNM in region VI (5.9%,9/153) was statistically significant higher than in other locations (0.0%, 0/382) (p<0.001), which is related to the anatomical proximity of LNM in region VI to the recurrent laryngeal nerve. Furthermore, univariate analysis and multivariate logistic regression analysis showed lymph node location in region VI was the independent risk factor for transient hoarseness, while size of LNM and multiple-time ablation was not.

For side effect, one patient experienced transient decrease in heart rate and blood pressure during ablation possibly due to vagus nerve stimulation, which was recovered after cessation of ablation. For minor complication, one patient experienced pain when lifting the right shoulder after ablation of two LNM in right region V, which was speculated caused by heat stimulation to accessory nerve. Furthermore, the symptoms were recovered spontaneously within 3 months without any sequelae. Importantly, there were no reports of local infection, hematoma, skin burn, trachea or esophagus perforation.

## Discussion

A systematic review revealed that LNM was present in 36.12% of PTC patients (21). Five to 20% of patients still experience new LNM after surgery (7). According to the American Thyroid Association, repeat surgery is recommended as the first-line treatment for LNM after surgery (6). However, reoperation due to postoperative fibrosis, tissue adhesion, and anatomical structure changes is associated with an increased risk of complications as well as technical challenges (8–10). Even for surgeons with extensive experience, the operation process for small- and medium-sized metastatic lymph nodes is challenging. The present retrospective study was conducted to investigate the efficacy and safety of MWA for LNM in initially treated, post-resection and post-ablation PTC patients, especially focusing on the risk of re-ablation or multiple time ablations.

In present study, the technique successful rate is 100%, regarding the LNM with different treating history- underwent resection, thermal ablation or untouched, as well as staged treatment in present study. The results emphasized that even with multiple ablation procedures, complete ablation could still be achieved in all the enrolled patients. However, repeated surgeries could be more challenging, resulting in increased complication and failure rates, as evidenced by higher rates of transient hypoparathyroidism in secondary surgery than in primary surgery (56.6% vs. 25.9%; p < 0.0001), permanent hypoparathyroidism (10% vs. 2.0%; p < 0.0001) and transient recurrent nerve injury (4.6% vs. 1.4%; p < 0.05) (10). Therefore, ablation is feasible for treating LNM.

In terms of effectiveness, throughout the mean follow-up period of 23.6 months, no local recurrence of LNM was observed, indicating that complete ablation of metastatic lymph nodes was achieved in all cases. According to our experience, the two key factors that may contribute to complete ablation are as follows. The first key factor is precise puncture guided by real-time ultrasound, which could guarantee the antenna tip puncture and stay inside the LNM during ablation, even the size of LNM is as small as several millimeter. The second factor is LNM capsule, which could limit heat conduction within LNM, to maintain a higher internal temperature inside LNM during ablation, potentially contributing to complete ablation. After ablation, the incidence of new LNM was 22.8% (39/171), which is comparable with the recurrence rate of 31.5% in PTC patients with LNM after total thyroidectomy during a mean follow-up period of 16.9 ± 0.6 years (22). There was no statistically significant difference among the initially treated, post-resection, and post-ablation subgroups. A long-term follow-up study revealed that after several surgical resections of LNM with persistent PTC lesions, 27% of patients were free of biochemical or clinical recurrences during a mean follow-up time of 60 months (23), which also indicated that the rate of new LNM is relatively low after treatment. In the present study, no distant metastases occurred during the follow-up period, which is comparable with the rate of 0.04% (1/2424) after surgery (24). Among the 32 patients with recurrent PTC, one (3.1%) died of thyroid cancer, which was a lower rate than the rate of 40.0% reported in a previous study (22). This patient was an elderly male patient who presented with a large sized metastatic lymph node, which was reported to be a high-risk factor for mortality in previous studies (22).

At the last follow-up after ablation, 84.1% (450/535) of the metastatic lymph nodes had completely disappeared, which is comparable with the rate of 80% reported in a meta-analysis (11), indicating that most metastatic lymph nodes could be absorbed after ablation.

For safety, transient hoarseness was the exclusive major complication with an incidence rate of 5.3% (9/171) in present study. For subgroup analysis, the rate was 8.0% (9/112) in the initially treated subgroup, which is comparable with the vocal cord paralysis rate of 8.3% reported in a comprehensive study of 2743 patients who underwent total thyroidectomy and unilateral lateral neck dissection (25). Furthermore, the rates were 0.0% (0/15) in the post-ablation subgroup and 0.0% (0/44) in the post-resection subgroup, both of which were markedly lower than the RLN injury incidence of 6.23% (20/321) reported in systematic research on thermal ablation for LNM of recurrent PTC (13). In addition, the overall incidence of transient hoarseness was not significantly different (p>0.5) among the patients who underwent one ablation procedure (5.3%, 7/131), two ablation procedures (5.5%, 9/164) and three ablation procedures (5.3%, 9/171), indicating that repeated ablations did not significantly increase the incidence of complications.

No other major complications were reported during the follow-up period. For side effect, one patient (0.8%, 1/131) experienced transient heart rate and blood pressure decreases during ablation of LNM in region IV. For minor complication, one patient (0.8%, 1/131) experienced pain when lifting the right shoulder after ablation of metastatic lymph nodes located in the right side of region V, of which recovered within 3 months.

In addition, hypoparathyroidism/hypocalcaemia occurred in 30.5% of patients after lateral neck dissection (25), whereas no such significant complications occurred in present study, suggesting that MWA could be considered a safer therapeutic option than conventional surgical resection.

The safety associated with MWA is attributed to several key factors. The application of continuous saline injection for hydrodissection, utilizing the specific heat capacity of water to control temperature increases effectively, was shown to further diminish the risk of complications. This approach also allowed for the creation of a protective buffer around the lesion, effectively distancing it from surrounding critical anatomical structures. Additionally, the use of high-frequency ultrasound (US) for real-time procedural guidance significantly enhanced the visualization of adjacent critical structures, thereby allowing for their avoidance and reducing the likelihood of procedure-related complications. The strategy of employing low-power (30W) and short-duration repeat ablation ensured that the temperature within the LNM remained elevated for effective ablation while keeping surrounding tissue temperatures lower, thus enhancing the overall safety of the MWA procedure.

These results highlight the potential of MWA as a safer, effective alternative for managing cervical LNM from PTC, with specific procedural strategies playing a crucial role in optimizing safety and treatment outcomes.

A few limitations were identified in this study. First, the present study is a retrospective study. Second, the study was characterized by a small sample size and a limited number of LNM that were subjected to MWA, indicating that further research with larger cohorts is urgently required to validate these findings. Third, a limited duration of follow-up was noted in this study. It is planned that continued monitoring of these patients will be carried out to obtain a more comprehensive understanding of the treatment outcomes.

## Conclusion

Microwave Ablation (MWA) is a feasible, effective, and safe treatment for LNM in initially treated, post-resection and postablation PTC patients, particularly with precision puncture and hydrodissection techniques, presenting a novel alternative for selected patients.

## Data Availability

The original contributions presented in the study are included in the article/supplementary material. Further inquiries can be directed to the corresponding author.
